# Outcome of Saphenous Vein Graft Percutaneous Coronary Intervention Using Contemporary Drug-Eluting Stents: A SCAAR Report

**DOI:** 10.1016/j.jscai.2024.102232

**Published:** 2024-09-26

**Authors:** Saman Saidi-Seresht, Stefan James, David Erlinge, Sasha Koul, Bo Lagerqvist, Moman Mohammad, Henrik Renlund, Per Grimfjärd

**Affiliations:** a Department of Cardiology, Västerås Hospital, Västerås, Sweden; b Department of Medical Sciences, Cardiology, Uppsala University, Uppsala, Sweden; c Department of Cardiology, Institute of Clinical Sciences, Lund University, Skane University Hospital, Lund, Sweden; d Uppsala Clinical Research Center, Uppsala, Sweden

**Keywords:** coronary artery bypass graft surgery, drug-eluting stent, percutaneous coronary intervention, saphenous vein graft

## Abstract

**Background:**

Percutaneous coronary intervention (PCI) of saphenous vein grafts (SVG) is associated with poor outcomes and is often regarded as inferior to native vessel PCI. We investigated clinical outcomes of SVG-PCI using contemporary drug-eluting stents (DES), in a complete, nationwide population.

**Methods:**

The complete Swedish Coronary Angiography and Angioplasty Registry (SCAAR) was used to identify all patients in Sweden who underwent SVG-PCI with a contemporary DES between 2013 and 2020. Baseline characteristics, procedures, and outcomes were described.

**Results:**

A total of 2198 SVG-PCI procedures with 3106 contemporary DES were included. Patients had a high incidence of comorbidities such as diabetes (40%), prior myocardial infarction (MI) (69%), and acute coronary syndrome (74%) at presentation. SVG-PCI procedures commonly involved multiple DES (41%). Native vessel PCI, in addition to SVG-PCI, was performed in only 13% of procedures. At 1 year, adverse clinical outcomes were frequent as exemplified by any death (9.2%), MI (9.1%), or revascularization (21.1%), whereas stent and lesion-related outcomes on a patient level were less common: stent thrombosis (1.2%), in-stent restenosis (4.3%) and target lesion revascularization (4.3%). Similarly, at 3 years, clinical outcomes were frequent: death (19.8%), MI (21.1%), revascularization (32.8%); and stent-related outcomes were less common: stent thrombosis (2.9%), restenosis (10.8), and target lesion revascularization (13.6%).

**Conclusions:**

In this nationwide cohort of patients who underwent SVG-PCI with contemporary DES, patients were characterized by a high-risk profile and high rates of adverse clinical events. However, the incidence of stent and lesion-related events was low.

## Introduction

Currently, 80% of coronary artery bypass graft surgery (CABG) procedures globally use saphenous vein grafts (SVG).^1^^,^^2^ Due to progressive vascular disease and SVG-specific pathophysiology, post-CABG patients commonly undergo percutaneous coronary intervention (PCI) of SVG, native-vessel PCI, and less commonly repeat CABG.^2^, ^3^, ^4^, ^5^, ^6^, ^7^, ^8^, ^9^, ^10^, ^11^ An SVG is targeted in 6% of PCI procedures in the United States.^3^ In relation to native vessel-PCI, SVG-PCI has often been regarded as inferior and associated with higher rates of peri-procedural and long-term adverse events including reintervention.^4^^,^^5^ A recent meta-analysis of observational studies in patients with prior CABG showed that compared with SVG-PCI, native vessel PCI had lower major adverse cardiac events (odds ratio 0.51, 95% CI 0.45-0.57, *P* < .001).^12^

Due to the high stent failure rate in SVG, PCI revascularization of a native coronary artery is advocated by many interventionists.^13^ However, this approach is yet to be proven superior in trials. The upcoming PROCTOR trial randomizes patients to SVG- or native vessel PCI.^14^ Albeit interesting, results will be difficult to apply universally, and individual considerations will remain important.

Post-CABG PCI predominantly targets native coronary arteries.^6^^,^^7^ Over time, most conduit PCI target SVG.^9^ With SVG failure, the choice between native vessel or SVG-PCI depends largely on native vessel lesion severity and degree of SVG degeneration.^6^^,^^15^ Although the outcome of SVG and native vessel PCI has improved with advances in drug-eluting stent (DES) technology, PCI methodology, and secondary prevention, many interventionists favor native vessel intervention. However, there is a lack of randomized data to guide such clinical decisions. The aim of this study was to describe outcome of SVG-PCI with contemporary DES in a complete national, high-risk, all-comer population.

## Methods

### The SCAAR registry

Swedish Coronary Angiography and Angioplasty Registry (SCAAR), a part of the national Swedish Web-system for Enhancement and Development of Evidence-based care in Heart Disease Evaluated According to Recommended Therapies (SWEDEHEART), covers data about all consecutive coronary angiographies and PCI procedures from all, 28, Swedish catheterization laboratory sites.^16^ Biweekly updates from the National Population Registry provide data on vital status and date of death.

### Study population

This analysis of the complete Swedish national population of patients undergoing PCI, from the 1st of January 2013 through the 31st of December 2019, included procedures where study stents were used. Study stents were defined as contemporary DES used in first-time stenting of de novo SVG lesions. The data includes 3106 study stents implanted in SVG in 2198 patients. During index procedures, in addition to study stents, some patients had 1 or several stents placed in the same SVG, another SVG, or a native coronary artery in 908 procedures. In 2013, 90% of all stents in SCAAR were drug-eluting, 100% from 2016.^2^ Only SVG-PCI was studied, and arterial graft PCI was excluded.

### Outcome measures

The rates of stent thrombosis (ST), in-stent restenosis (ISR), target lesion revascularization (TLR), and target graft revascularization were analyzed on both stent and procedure (patient) levels, with 1- and 3-year follow-up, the end of study period or death. Stent level and procedure (patient) level analysis refers to the 2 main different ways of using the SCAAR dataset. Procedure (patient) level analysis is based on each individual patient being subject to PCI and meaningful outcomes include death, myocardial infarction (MI), and revascularization. Stent level analysis is based on each individual stent. Stents can be followed up one by one for stent-related outcomes like ST and ISR, thus enabling more detailed data on stent performance, because many patients have more than 1 stent implanted. The fourth universal MI definition and ST definitions by the ARC were applied.^8^^,^^17^ ISR is defined as significant stenosis in a previously stented segment. Bleeding definitions are listed in Supplemental Table S1.

### Statistical analyses

All analyses were carried out using R (The R Foundation). Follow-up times were calculated from procedure to event, death, or end of follow-up. The limit was 31st of December 2020. The mean follow-up time, defined as the time between the index procedure and death (if any), was 3.6 years with a standard deviation of 2 years. All-cause mortality and MI rates were analyzed on a patient level and the remaining end points were analyzed on a patient and stent level. All outcomes were counted and censored by death. ST and ISR censored each other. Stent level outcome for study stents was stratified by solitary stents, nonsolitary stents, and all combined. Solitary stent was defined as a single study stent implanted in 1 vein-graft and nonsolitary stents as more than 1 stent implanted in the same vein-graft. Patient-level outcomes were stratified by single and multiple stent procedures, and all combined. A single stent procedure had only 1 solitary stent, whereas multiple stent procedures may have had 1, or several such stents, with or without stents in a native vessel.

## Results

The data include 3106 stents implanted in SVG in 2198 post-CABG patients. On the patient level, the median age was 74 years with male predominance. ACS was the indication in 74% of PCI with a high prevalence of risk factors: 45% had previous PCI, 69% had previous MI, and 40% had diabetes. Baseline characteristics were similar in patients receiving single and multiple stents (Table 1) as well as in patients with diabetes (Supplemental Table S2).

**Table 1:** Patient characteristics.

Variable	Single stent (n = 1290)	Multiple stents (n = 908)	All procedures (N = 2198)
Age, years	74.3 (68.5-79.8) [0]	73.8 (68.9-79.4) [0]	74.2 (68.7-79.7) [0]
STEMI	12.3% (159)	11.6% (105)	12% (264)
Unstable angina	13.6% (175)	12.7% (115)	13.2% (290)
Non-STEMI	50.1% (646)	47.9% (435)	49.2% (1081)
Stable angina	20.5% (265) [0]	24% (218) [0]	22% (483) [0]
Other indications	3.5% (45)	3.9% (35)	3.6% (80)
Previous myocardial infarction	68.1% (844) [50]	70.7% (629) [18]	69.2% (1473) [68]
Previous coronary angioplasty	42.7% (550) [1]	47.8% (433) [2]	44.8% (983) [3]
Diabetes, insulin-treated	21.8% (279) [8]	21.5% (195) [3]	21.7% (474) [11]
Diabetes, noninsulin treated	17.6% (225)	18.2% (165)	17.8% (390)
Female sex	14.7% (189) [0]	12.7% (115) [0]	13.8% (304) [0]
Body mass index, kg/m^2^	27.2 (24.8-30) [79]	27.3 (24.8-30.1) [48]	27.2 (24.8-30.1) [127]
Creatinine, μmol/L	89 (75-110) [222]	90 (77-109) [167]	90 (76-109) [389]
Hypertension	89.3% (1146) [7]	91.6% (827) [5]	90.3% (1973) [12]
Hyperlipidemia	92% (1180) [8]	90.8% (822) [3]	91.5% (2002) [11]
Never smoked	35% (422) [83]	35.8% (307)[50]	35.3% (729) [133]
Former smoker	53.8% (649)	54.9% (471)	54.2% (1120)
Current smoker	11.3% (136)	9.3% (80)	10.5% (216)

Continuous variables: median (Q1-Q3). Categorical variables: Percentage (count). [n] is missing count.

STEMI, ST-elevation myocardial infarction.

Nearly 96% of patients had multivessel disease and 27% had left main disease. The most common index PCI target was an LCX conduit followed by an RCA conduit and an LAD conduit. Average lesion complexity was high and thrombectomy was uncommon. The most common DES were Resolute Onyx (Medtronic), Synergy (Boston Scientific), Promus Premier (Boston Scientific), and Xience platform (Abbott), constituting 27.2%, 24.3%, 20.5%, and 12%, respectively (Supplemental Table S3). In ∼ 60% of index procedures, a single DES was implanted in an SVG and ∼ 40% involved more than 1 DES. SVG-only PCI was performed in 87% while a native vessel was stented in 13% of all procedures (data not shown). The operator reported success was 98% (Table 2). Complication rates were low, and no procedure-related death was reported (Supplemental Table S4**)**.

**Table 2:** Extent of coronary artery disease and procedure characteristics.

Variable	Single stent (n = 1290)	Multiple stents (n = 908)	All procedures (N = 2198)
3-vessel disease, not left main	50.6% (653)	55.3% (502)	52.5% (1155)
2-vessel disease, not left main	15.6% (201)	14.8% (134)	15.2% (335)
1-vessel disease, not left main	5.2% (67)	3.3% (30)	4.4% (97)
Left main	27.5% (355)	25.9% (235)	26.8% (590)
SVG to LCX treated	46.7% (602) [0]	54.7% (497) [0]	50% (1099) [0]
SVG to RCA treated	34.8% (449) [0]	42.1% (382) [0]	37.8% (831) [0]
SVG to LAD treated	21.9% (282) [0]	23.5% (213) [0]	22.5% (495) [0]
Any B2/C lesion treated	60.1% (775) [0]	75% (681) [0]	66.2% (1456) [0]
Any bifurcation (B1/B2/C) treated	2.6% (34) [0]	8.9% (81) [0]	5.2% (115) [0]
Any chronic occlusion treated	2.6% (33) [1]	6.5% (59) [1]	4.2% (92) [2]
Any LAD lesion treated	0.78% (10) [0]	6.6% (60) [0]	3.2% (70) [0]
Any LCX lesion treated	1.5% (19) [0]	13.4% (122) [0]	6.4% (141) [0]
Any LM lesion treated	0.16% (2) [0]	6.2% (56) [0]	2.6% (58) [0]
Any RCA lesion treated	1% (13) [0]	11% (100) [0]	5.1% (113) [0]
Any OAC treatment before procedure	10.9% (141) [0]	9.6% (87) [0]	10.4% (228)[0]
Gp IIb/IIIa inhibitor treatment during procedure	4.2% (54) [0]	5.3% (48) [0]	4.6% (102) [0]
Multivessel coronary angioplasty	3.3% (43) [0]	33.3% (302) [0]	15.7% (345) [0]
Thrombectomy performed	5.1% (66) [0]	7.3% (66) [0]	6% (132) [0]
Mean diameter of stents, mm	3.5 (3-3.5) [0]	3.2 (2.9-3.5) [0]	3.5 (3-3.5) [0]
Total length, mm	18 (16-24) [0]	22 (16-32) [0]	20 (18-45) [0]
Max pressure for stent, atm	19 (16-20) [7]	20 (16-20) [3]	20 (16-20) [10]
Radial access	48.5% (625) [0]	48.4% (440) [0]	48.5% (1065) [0]
Femoral access	50.2% (648) [0]	49.2% (447) [0]	49.8% (1095) [0]
Radial and femoral access	1.1% (14) [0]	2.2% (20) [0]	1.5% (34) [0]
Procedure success	98.3% (1268) [0]	98.2% (892) [0]	98.3% (2160) [0]

Continuous variables: median (Q1-Q3). Categorical variables: percentage (count). [n] is missing count.

Gp, glycoprotein; LAD, left anterior descending artery; LCX, left circumflex artery; LM, left main; OAC, oral anticoagulant; RCA, right coronary artery; SVG, saphenous vein graft.

**Figure 1:**
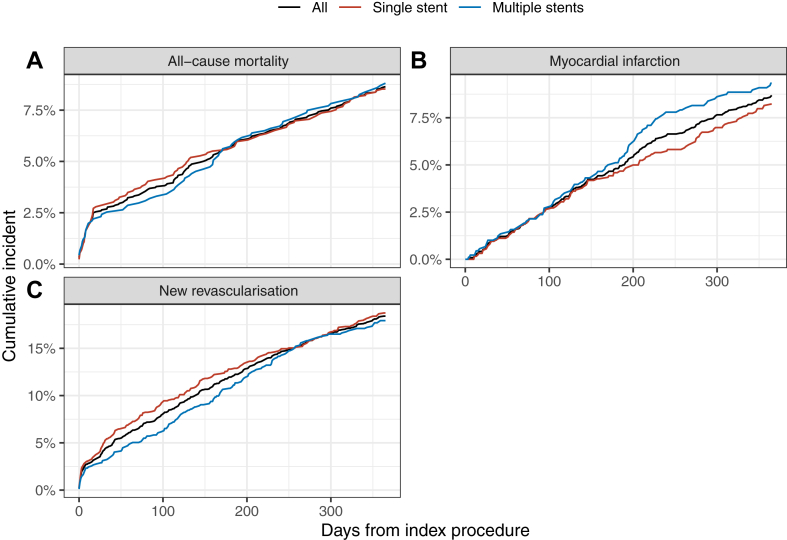
**One-year patient-level outcome.** Percentages, not Kaplan-Meier estimates. (**A**) All-cause mortality, (**B**) myocardial infarction, (**C**) new revascularization.

**Figure 2:**
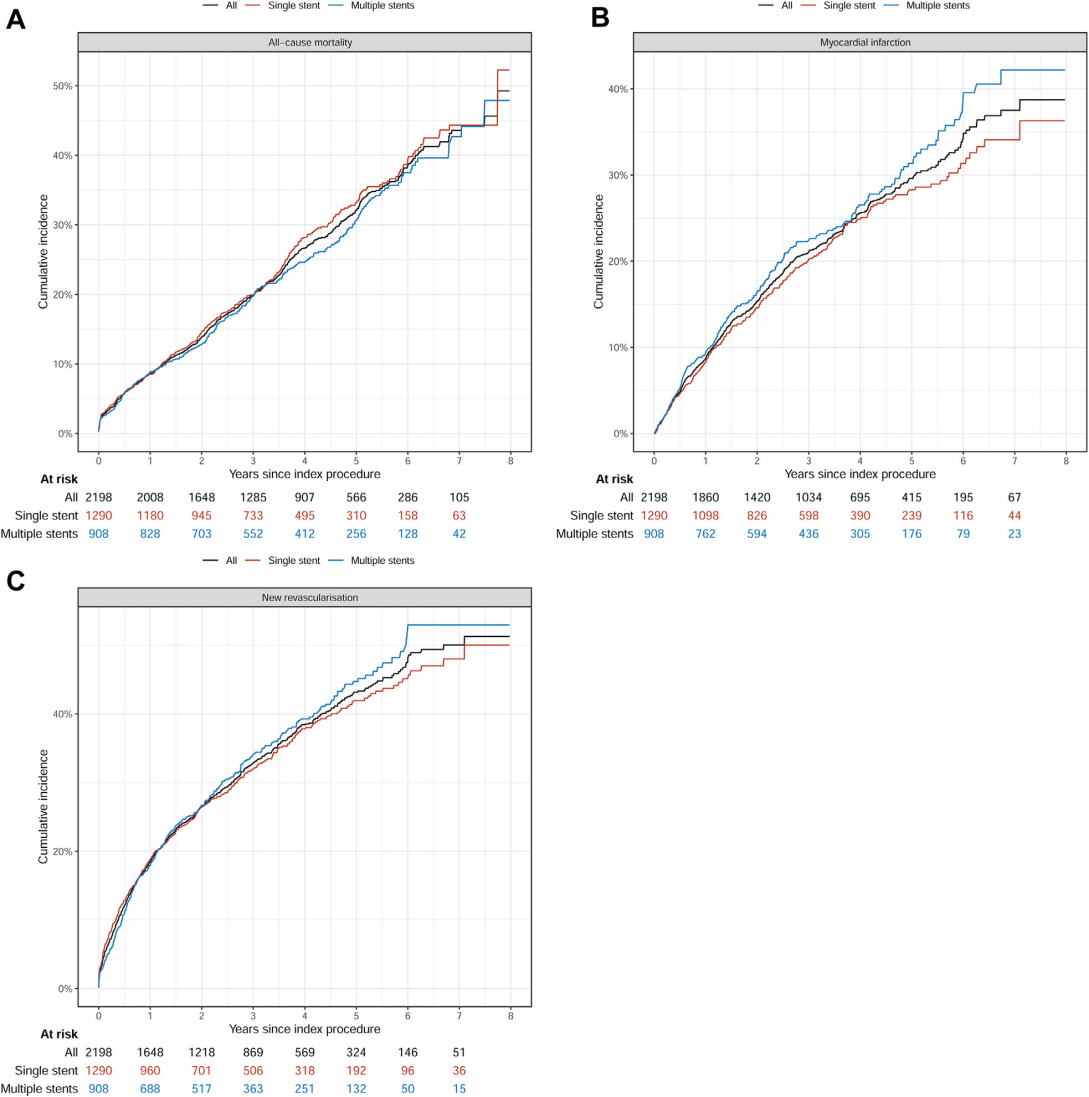
**Total study period patient-level outcome.** Kaplan-Meier curves of cumulative incidence. (**A**) All-cause mortality, (**B**) myocardial infarction, (**C**) new revascularization.

**Figure 3:**
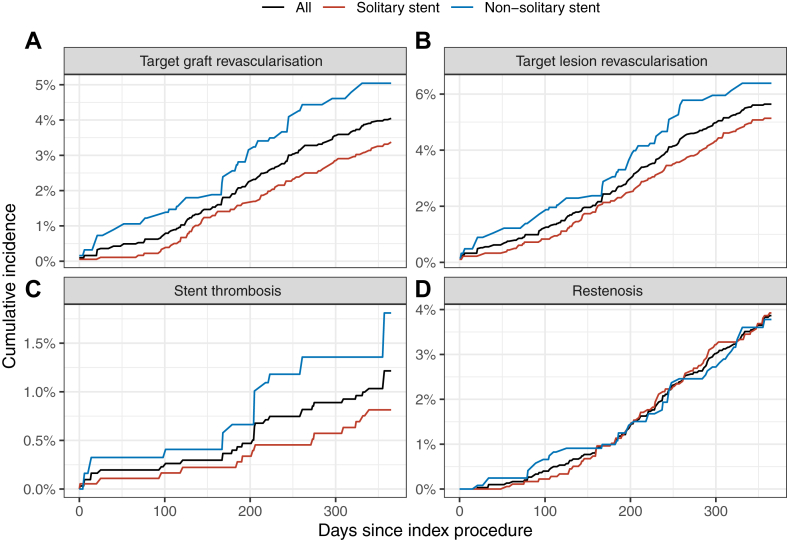
**One-year stent level outcome.** Percentages, not Kaplan-Meier estimates. (**A**) Target graft revascularization, (**B**) target lesion revascularization, (**C**) stent thrombosis, (**D**) restenosis.

**Figure 4:**
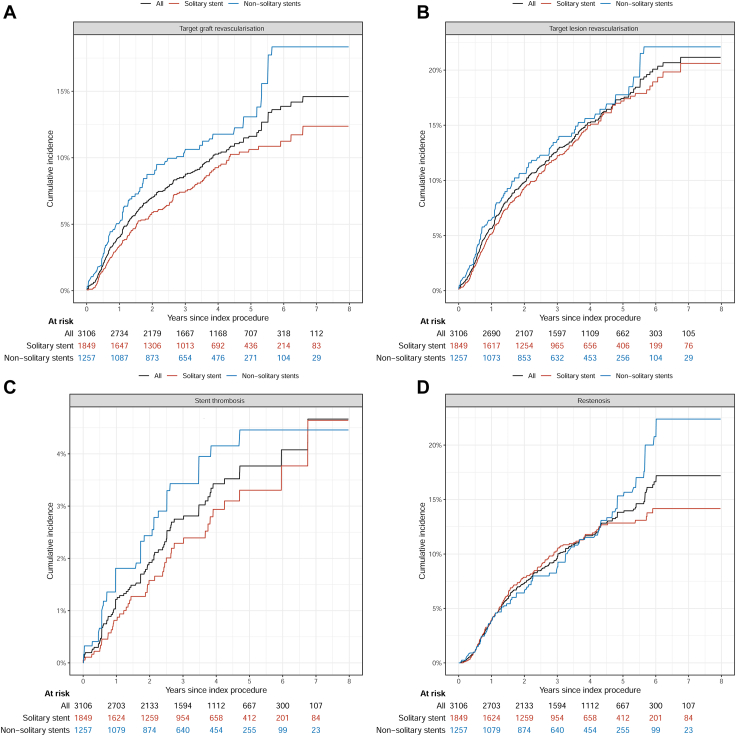
**Total study period stent level outcome.** One-year stent-level outcomes. Percentages, not Kaplan–Meier estimates. (**A**) Target graft revascularization, (**B**) target lesion revascularization, (**C**) stent thrombosis, (**D**) restenosis.

All outcomes are presented in Figure 1, Figure 2, Figure 3, Figure 4 and Tables 3 and 4. Adverse clinical outcomes were frequent: at 1 year, death was 9.2%, MI 9.1%, and TLR was 4.3%; at 3 years, death was 19.8%, MI 21.1%, and TLR was 13.6%, respectively. Stent-related adverse events were less common: at 1 year, ST was 1.2% and ISR was 4.3%; at 3 years, ST was 2.9% and ISR was 10.8%. Diabetes was generally associated with higher adverse outcome rates (Supplemental Tables S5 and S6).

**Table 3:** One-year outcome, all patients.

Variable	Time	Events	Rate	Time	Events	Rate	Time	Events	Rate
Stent-level outcomes									
	Solitary			Non-solitary			All		
Stent thrombosis	1724	14	0.8	1159	21	1.8	2883	35	1.2
In-stent restenosis	1724	68	3.9	1159	44	3.8	2883	112	3.9
Target lesion revascularization	1729	59	3.4	1153	60	5.2	2882	119	4.1
Target graft revascularization	1714	90	5.2	1145	76	6.6	2860	166	5.8
Patient-level outcomes									
	Single stent			Multiple stents			All		
Stent thrombosis	1198	13	1.1	839	11	1.3	2037	24	1.2
In-stent restenosis	1198	46	3.8	839	42	5.0	2037	88	4.3
Target lesion revascularization	1200	44	3.7	839	44	5.2	2039	88	4.3
Target graft revascularization	1188	69	5.8	834	56	6.7	2022	125	6.2
All-cause mortality	1218	110	9.0	859	80	9.3	2076	190	9.2
Myocardial infarction	1171	101	8.6	820	81	9.9	1991	182	9.1
Any revascularization	1070	231	22.4	772	156	20.2	1843	387	21.1

Outcome rates at 1 year, follow-up time in patient-years (n), events (n), and rate (%).

**Table 4:** Three-year outcomes, all patients.

Variable	Time	Rate	Time	Rate	Time	Rate
Stent-level outcomes						
	Solitary		Non-solitary		All	
Stent thrombosis	954	2.3	642	3.4	1594	2.8
In-stent restenosis	954	10.5	642	8.5	1594	9.7
Target lesion revascularization	1013	7.5	655	10.4	1667	8.7
Target graft revascularization	965	12.2	633	13.4	1597	12.7
Patient-level outcomes						
	Single stent		Multiple stents		All	
Stent thrombosis	646	2.3	469	3.8	1115	2.9
In-stent restenosis	646	9.6	469	12.4	1115	10.8
Target lesion revascularization	652	12.1	470	15.7	1121	13.6
Target graft revascularization	682	7.6	497	10.9	1178	9.0
All-cause mortality	734	19.9	553	19.7	1286	19.8
Myocardial infarction	599	20.2	436	22.4	1034	21.1
Any revascularization	506	32.1	364	33.9	869	32.8

Kaplan-Meier estimates at 3 years, follow-up time in patient-years (n), rate (%).

## Discussion

This study shows favorable stent-related SVG-PCI outcomes with contemporary DES whereas the risk of patient-related adverse clinical outcomes remains high in the context of this extremely high-risk profile population (Central Illustration).

**Central Illustration:**
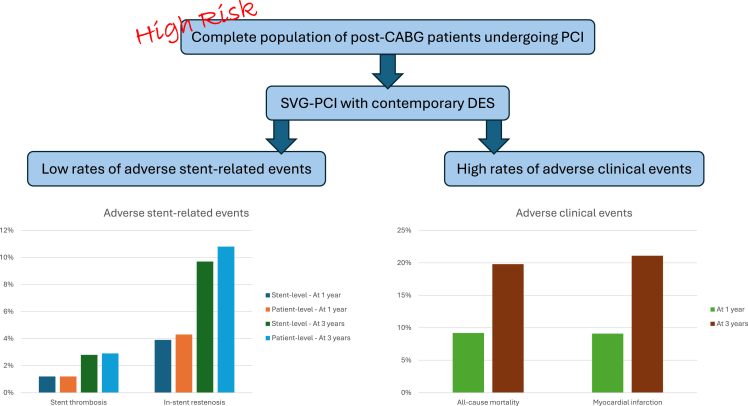
The outcome of saphenous vein graft (SVG) percutaneous coronary intervention (PCI) using contemporary drug-eluting stents (DES). CABG, coronary artery bypass graft surgery.

An important aspect of this study is that contemporary data from a complete, national, all-comer population on the outcome of de novo SVG-PCI using contemporary DES have been analyzed. Reassuringly, the rates of periprocedural complications were low and no procedure-related deaths were recorded despite the given high-risk population background.

Interestingly, although the evidence for the superiority of DES over BMS in SVG-PCI is conflicting,^3^^,^^18^, ^19^, ^20^, ^21^, ^22^, ^23^, ^24^, ^25^, ^26^, ^27^ almost all current SVG-PCI are treated with DES.

In this study, the 1-year stent-level ST and ISR rates were 1.2% and 3.9%, respectively compared with 1.9% and 7.5% in a similar SCAAR report on SVG-PCI a decade ago.^28^ The corresponding rates for ST and ISR at 3 years were 2.8% and 9.7% in this study whereas they were ∼ 4% and 20% in the former SCAAR report, suggesting substantial improvement in long-term stent outcome with contemporary DES. Because the threshold to subject a post-CABG patient to angiography has decreased over the last decade, the observed improvements in rates of ST and ISR are likely underappreciated.

The patient-level 3-year ISR rate of 10.8% in this study was comparable with a recent meta-analysis of SVG-PCI with an ISR rate of 9.4% over 2.7 years average follow-up.^5^

In the current stent-level analysis, the rates of ISR were similar between single and multiple stent groups at 1 year but higher in the single stent group at 3 years. This may be a result of undetermined bias or competing risk.

As compared to previous international reports on SVG-PCI, this study also showed a lower 1-year stent-level target graft revascularization compared to studies of mixed stents (DES and bare metal stents) and first-generation DES (5.8% vs 9% and 11%).^18^^,^^29^ The current patient-level rates of ST (2.9%) and ISR (10.8%) at 3 years are high, as expected, compared with 2 recent all-comer studies of any-vessel-PCI with contemporary DES and 2 to 3 years follow-up, both demonstrating ST and ISR rates of roughly 0.8% and 2%, respectively.^30^^,^^31^

The alternative to SVG-PCI often involves recanalization of chronic total occlusions which carried a TLR rate of 14% over 4 years in 1 trial.^32^ Counterintuitively for many interventionists, the patient-level TLR rate at 3 years, in this study, was “only” 13.6%.

The patient-level TLR of 15.1% in patients with diabetes and 4.3% for all patients at 1 year may be put in perspective with corresponding rates in a trial of recanalized chronic total occlusions in patients with and without diabetes with a TLR of 7.2% and 4.5%, respectively.^33^ In comparison, in a recent all-comers study of newer-generation DES, the TLR rate at 2 years was about 1.4%.^31^

Similar to the outcome of multivessel-PCI in native coronary arteries,^34^^,^^35^ multiple stents in the current trial increased the risk of stent-related outcomes. However, death rates remained similar regardless of stent burden, reflecting the high-risk profile of the studied population which translates into an overall very high clinical event rate. In fact, one-fifth of patients died, one-fifth experienced a new MI and one-third underwent a new revascularization procedure in 3 years. A recent meta-analysis of SVG-PCI with ∼ 3 years follow-up also reported a high major adverse cardiac events rate (death, MI, and target vessel revascularization) of 35%.^5^

In this study, diabetes was generally associated with higher adverse event rates. This was more evident for death, MI, and revascularization than stent-related events like ST, ISR, and TLR, indicating that diabetic patient-intrinsic factors have more pronounced effects on hard outcomes rather than SVG-stent performance.

Further research is required to optimize the short and long-term outcome of this high-risk population as well as determine if native vessel PCI should be the preferred option after SVG failure.

### Limitations

This study reports descriptive data on patient and procedural characteristics and outcome after SVG-PCI using a nationwide cohort of patients. No suitable comparison arm, with similar baseline risk, has been identified in the SCAAR dataset; hence, outcome data are compared to historical data. Native vessel PCI, in addition to SVG-PCI, in 13% of cases may have an impact on results. TLR, ST, ISR are operator reported events and nonadjudicated or core-lab evaluated. However, regular assessments are performed on a subset of patients to ensure source data adherence and reporting. The latest large evaluation showed 97% data consistency in reporting between sites (not published). In SWEDEHEART, all recorded events are clinically driven. Hence, there is no active safety surveillance, and asymptomatic outcome events may not be captured. Our data lack specific information on the use of imaging and embolic protection devices, which may potentially impact outcomes.

## Conclusions

This national, complete, all-comer, high-risk population of patients undergoing SVG-PCI with contemporary DES is associated with high rates of adverse events. Compared with similar data from the last decade, this study suggests improvement of stent and lesion-related outcomes after SVG-PCI using contemporary DES.
