# Supplementary material for: Outcome of Saphenous Vein Graft Percutaneous Coronary Intervention Using Contemporary Drug-Eluting Stents: A SCAAR Report
Source: J Soc Cardiovasc Angiogr Interv. 2024 Sep 26;3(10):102232. doi: 10.1016/j.jscai.2024.102232 (PMC11549509; doi:10.1016/j.jscai.2024.102232)
Supplement: Supplementary Tables [file mmc1.docx]

**Supplements**

**Supplemental Table S1. Bleeding definitions**

| Major bleeding | Intracranial haemorrhage or any other bleeding leading to haemoglobin (Hb) drop of more than 50 g/L. |
| --- | --- |
| Minor bleeding | Hb drop more than 30 g/L but less than 50 g/L. |
| Any bleeding | Both major and minor bleeds plus bleeding requiring more than simple compression treatment or prolongs hospital stay. |

**Supplemental Table S2. Diabetic subgroup characteristics**

| **Variable** | **Single stent**  n=1290 | **Multiple stent**  n=908 | **All**  n=2198 |
| --- | --- | --- | --- |
| Age | 73.5 (67.6 - 78.3)[0] | 73.3 (68.5 - 78.8)[0] | 73.4 (67.8 - 78.5)[0] |
| STEMI | 10.7% (54) | 9.7% (35) | 10.3% (89) |
| Unstable angina | 13.5% (68) | 10% (36) | 12% (104) |
| Non-STEMI | 53.4% (269) | 51.7% (186) | 52.7% (455) |
| Chronic coronary artery disease | 18.8% (95)[0] | 24.2% (87)[0] | 21.1% (182)[0] |
| Indication: Other | 3.6% (18) | 4.4% (16) | 3.9% (34) |
| Previous myocardial infarction | 73.5% (357)[18] | 74.4% (265)[4] | 73.9% (622)[22] |
| Previous coronary angioplasty | 43.8% (221)[0] | 53.6% (193)[0] | 47.9% (414)[0] |
| Diabetes, insulin treated | 55.4% (279)[0] | 54.2% (195)[0] | 54.9% (474)[0] |
| Diabetes, non-insulin treated | 44.6% (225) | 45.8% (165) | 45.1% (390) |
| Gender: Female | 15.5% (78)[0] | 15.3% (55)[0] | 15.4% (133)[0] |
| BMI (kg/m2) | 28.4 (25.6 - 31.2)[24] | 28.4 (25.6 - 31.6)[20] | 28.4 (25.6 - 31.4)[44] |
| Creatinine (µmol/L) | 91 (76 - 116.2)[80] | 93 (76 - 117)[57] | 91 (76 - 116.5)[137] |
| Hypertension | 93.3% (470)[0] | 95.5% (342)[2] | 94.2% (812)[2] |
| Hyperlipidaemia | 94.8% (476)[2] | 95.5% (343)[1] | 95.1% (819)[3] |
| Never smoker | 31.3% (151)[21] | 33.9% (115)[21] | 32.4% (266)[42] |
| Former smoker | 56.1% (271) | 57.5% (195) | 56.7% (466) |
| Current smoker | 12.6% (61) | 8.6% (29) | 10.9% (90) |

Numeric variables: median (Q1-Q3). Category variables: Percentage (count). [n] is missing count.

STEMI=ST-elevation myocardial infarction.

**Supplemental Table S3. Stents included**

| **Stent** | **Solitary** | **Non-Solitary** | **All** |
| --- | --- | --- | --- |
| Abbot Xience Sierra | 0.38% (7) | 0.4% (5) | 0.39% (12) |
| Abbott Xience Alpine | 1.1% (21) | 1.5% (19) | 1.3% (40) |
| Abbott Xience Prime | 0.22% (4) | 0.24% (3) | 0.23% (7) |
| Abbott Xience ProA | 0.65% (12) | 1.4% (17) | 0.93% (29) |
| Abbott Xience ProX | 3.2% (60) | 3.1% (39) | 3.2% (99) |
| Abbott Xience V | 0.054% (1) | 0% (0) | 0.032% (1) |
| Abbott Xience Xpedition | 6.4% (118) | 4.9% (62) | 5.8% (180) |
| Biotronik Orsiro | 9.9% (183) | 11.5% (145) | 10.6% (328) |
| BS Promus Elite | 2.2% (41) | 1.4% (18) | 1.9% (59) |
| BS Promus Premier | 21.8% (403) | 18.5% (233) | 20.5% (636) |
| BS Synergy | 22.7% (420) | 26.7% (336) | 24.3% (756) |
| Medtronic Resolute Onyx | 27.6% (511) | 26.6% (334) | 27.2% (845) |
| Terumo Ultimaster | 3.7% (68) | 3.7% (46) | 3.7% (114) |

Category variables: percentage (count).

Abbott Xience family: Abbott group, Biotronic Orsiro: Biotronic group, BS family: Boston scientific (BS) corporation, Medtronic Resolute Onyx: Medtronic PLC, Terumo Ultimaster: Terumo corporation.

**Supplemental Table S4. Complications**

| **Variable** | **Single stent**  n=1290 | **Multiple stent**  n=908 | **All**  n=2198 |
| --- | --- | --- | --- |
| Bleeding, major | 0% (0) | 0.34% (3) | 0.14% (3) |
| Bleeding requiring surgical procedure | 0.079% (1) | 0.11% (1) | 0.092% (2) |
| Required other treatment in addition to compression | 0.16% (2) | 0.11% (1) | 0.14% (3) |
| Pseudoaneurysm requiring treatment | 0.079% (1) | 0.11% (1) | 0.092% (2) |
| Any complication reported from the ward | 4.7% (60) | 5.9% (53) | 5.2% (113) |
| Procedure-related death in the ward | 0% (0) | 0% (0) | 0% (0) |
| Any complication reported from the lab | 1.4% (18) | 2.3% (21) | 1.8% (39) |
| Extended hospital stay *>*1 day | 0.79% (10) | 0.67% (6) | 0.74% (16) |
| Acute arrhythmia with urgent treatment needed | 0.39% (5) | 0.44% (4) | 0.41% (9) |
| Perforation | 0.31% (4) | 0.77% (7) | 0.5% (11) |
| Side-branch closure | 0.078% (1) | 0.22% (2) | 0.14% (3) |
| Hemodynamic complication | 0.16% (2) | 0.55% (5) | 0.32% (7) |
| Reinfarction during index hospital admission | 0.47% (6) | 1.3% (12) | 0.83% (18) |
| Non-coronary vascular complication | 0.16% (2) | 0.11% (1) | 0.14% (3) |

Percentage (count). Hb=Haemoglobin, CABG= coronary artery bypass graft.

**Supplemental Table S5.** One-year outcome, patients with diabetes.

| Variable | Time | Events | Rate | Time | Events | Rate | Time | Events | Rate |
| --- | --- | --- | --- | --- | --- | --- | --- | --- | --- |
| Stent-level outcomes | | | | | | | | | |
|  | Solitary | | | Nonsolitary | | | All | | |
| Stent thrombosis | 677 | 8 | 1.2 | 449 | 11 | 2.4 | 1126 | 19 | 1.7 |
| In-stent restenosis | 677 | 30 | 4.4 | 449 | 17 | 3.8 | 1126 | 47 | 4.2 |
| Target lesion revascularization | 676 | 28 | 4.1 | 447 | 26 | 5.8 | 1123 | 54 | 4.8 |
| Target graft revascularization | 670 | 43 | 6.4 | 445 | 32 | 7.2 | 1115 | 75 | 6.7 |
| Patient-level outcomes | | | | | | | | | |
|  | Single stent | | | Multiple stents | | | All | | |
| Stent thrombosis | 466 | 7 | 1.5 | 327 | 6 | 1.8 | 793 | 13 | 1.6 |
| In-stent restenosis | 466 | 18 | 3.9 | 327 | 19 | 5.8 | 793 | 37 | 4.7 |
| Target lesion revascularization | 463 | 21 | 4.5 | 327 | 19 | 5.8 | 790 | 40 | 5.1 |
| Target graft revascularization | 458 | 35 | 7.6 | 326 | 22 | 6.8 | 784 | 57 | 7.3 |
| All-cause mortality | 474 | 48 | 10.0 | 335 | 41 | 12.0 | 809 | 89 | 11.0 |
| Myocardial infarction | 450 | 55 | 12.0 | 315 | 43 | 14.0 | 765 | 98 | 13.0 |
| Any revascularization | 407 | 105 | 26.0 | 299 | 67 | 22.0 | 706 | 172 | 24.0 |

Outcome rates at 1 year; time refers to total patient-years follow-up (n), events (n), and rate (%).

**Supplemental Table S6.** Three-year outcomes, patients with diabetes.

| Variable | Time | Rate | Time | Rate | Time | Rate |
| --- | --- | --- | --- | --- | --- | --- |
| Stent-level outcomes | | | | | | |
|  | Solitary | | Nonsolitary | | All | |
| Stent thrombosis | 350 | 2.6 | 237 | 3.8 | 586 | 3.0 |
| In-stent restenosis | 350 | 12.6 | 237 | 9.1 | 586 | 11.2 |
| Target lesion revascularization | 378 | 8.5 | 247 | 10.5 | 624 | 9.3 |
| Target graft revascularization | 355 | 13.7 | 239 | 13.9 | 593 | 13.8 |
| Patient-level outcomes | | | | | | |
|  | Single stent | | Multiple stents | | All | |
| Stent thrombosis | 243 | 2.8 | 166 | 3.5 | 409 | 3.1 |
| In-stent restenosis | 243 | 9.7 | 166 | 16.2 | 409 | 12.4 |
| Target lesion revascularization | 242 | 13.0 | 169 | 18.1 | 410 | 15.1 |
| Target graft revascularization | 257 | 8.2 | 180 | 12.8 | 436 | 10.1 |
| All-cause mortality | 279 | 22.4 | 201 | 24.9 | 479 | 23.5 |
| Myocardial infarction | 221 | 23.4 | 140 | 31.4 | 360 | 26.7 |
| Any revascularization | 186 | 34.4 | 120 | 40.1 | 305 | 36.7 |

Kaplan-Meier estimates at 3 years; time refers to total patient-years follow-up (n), rate (%).
